# Metal Dyshomeostasis and Inflammation in Alzheimer's and Parkinson's Diseases: Possible Impact of Environmental Exposures

**DOI:** 10.1155/2013/726954

**Published:** 2013-04-17

**Authors:** Oddvar Myhre, Hans Utkilen, Nur Duale, Gunnar Brunborg, Tim Hofer

**Affiliations:** ^1^Division of Environmental Medicine, Department of Chemicals and Radiation, The Norwegian Institute of Public Health, P.O. Box 4404, Nydalen, 0403 Oslo, Norway; ^2^Division of Environmental Medicine, Department of Food, Water and Cosmetics, The Norwegian Institute of Public Health, P.O. Box 4404, Nydalen, 0403 Oslo, Norway

## Abstract

A dysregulated metal homeostasis is associated with both Alzheimer's (AD) and Parkinson's (PD) diseases; AD patients have decreased cortex and elevated serum copper levels along with extracellular amyloid-beta plaques containing copper, iron, and zinc. For AD, a putative hepcidin-mediated lowering of cortex copper mechanism is suggested. An age-related mild chronic inflammation and/or elevated intracellular iron can trigger hepcidin production followed by its binding to ferroportin which is the only neuronal iron exporter, thereby subjecting it to lysosomal degradation. Subsequently raised neuronal iron levels can induce translation of the ferroportin assisting and copper binding amyloid precursor protein (APP); constitutive APP transmembrane passage lowers the copper pool which is important for many enzymes. Using *in silico* gene expression analyses, we here show significantly decreased expression of copper-dependent enzymes in AD brain and metallothioneins were upregulated in both diseases. Although few AD exposure risk factors are known, AD-related tauopathies can result from cyanobacterial microcystin and **β**-methylamino-L-alanine (BMAA) intake. Several environmental exposures may represent risk factors for PD; for this disease neurodegeneration is likely to involve mitochondrial dysfunction, microglial activation, and neuroinflammation. Administration of metal chelators and anti-inflammatory agents could affect disease outcomes.

## 1. Introduction

Degenerative brain disorders among the elderly constitute a growing burden for the affected individuals, their families, and for the society. The mechanisms behind Alzheimer's and Parkinson's diseases (AD and PD) are still unclear. Although symptom-relieving drugs exist, drugs halting their progression are still lacking [1–5]. Our brains are continuously exposed to a broad spectrum of chemicals from various sources; these may react or interfere with biomolecules, or they may bioaccumulate (e.g., mercury, nanoparticles, and pesticides and persistent organic pollutants (POPs)), causing changes in brain function. In addition, an endogenous production of metabolic byproducts including free radicals inflict damages which constantly need to be repaired. Whereas repeated single chemical exposures may be associated with PD [6–8], convincing evidence is lacking that single chemical exposures (e.g., copper, mercury, or POPs) are causal for AD; several previously suspected agents (e.g., aluminium from antiperspirants and pans) have been written off [9]. However, there is strong support for the involvement of metal dyshomeostasis, inflammation, and oxidative damages to biomolecules in both diseases [10–13]. AD is a slowly progressing disease for which age is the most important risk factor [1, 2], and factors regulating metal homeostasis and inflammation known to change with age may be causally related to both AD and PD. Microglia activation is seen in multiple sclerosis [14], stroke [15], traumatic brain injury [16], Creutzfeldt-Jakob disease [17], in addition to PD and AD.

In this paper, AD metal dyshomeostasis and potential AD-causative cyanobacterial neurotoxins present in water and foods are discussed. We propose a neuronal metal dyshomeostasis mechanism mediated by the systemic iron regulatory peptide hepcidin and discuss therapeutic use of metal chelators and possible preventive measures. For PD, putative roles of chemical and nanoparticle exposure, metal dyshomeostasis and inflammation are discussed.

## 2. Alzheimer's Disease (AD)

**Figure 1:**
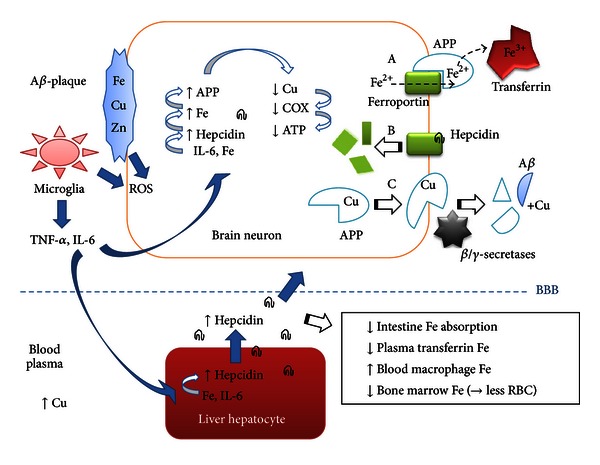
Putative linkage between cytokine (IL-6) and iron (Fe) induced hepcidin production with APP-mediated copper (Cu) lowering in the AD brain. Both liver and brain cells can produce the iron regulatory peptide hepcidin which may cross the blood-brain barrier (BBB). (A) In neurons, plasma membrane localized ferroportin exports ferrous iron (Fe^2+^) which is oxidized extracellularly by means of the ferroportin-collaborating amyloid precursor protein (APP) which has ferroxidase activity [18] and which loads ferric iron (Fe^3+^) into transferrin. (B) Hepcidin binding to ferroportin causes its internalization and lysosomal breakdown, preventing iron export [19, 20]. In response, iron levels in individual neurons may increase during aging, initiating APP-mRNA iron responsive constitutive translation of APP [21] which contains a copper binding domain. (C) APP travels to the plasma membrane and is cleaved by secretases to form short peptides, of which the A*β* peptide can form plaques containing metal ions [22–24]. Lowered neuronal copper levels, for which the cellular pool is low, can affect vital copper enzymes negatively (e.g., mitochondrial respiratory ATP producing COX, Cu/Zn-SOD, etc.). A*β*-plaque attacking microglia release various cytokines including IL-6 and ROS, that along with ROS generated from A*β*-plaque associated redox-cycling metals (e.g., Fe and Cu ions) inflict free radical damage to neurons. Liver hepcidin production affects iron metabolism in several organs (see text).

AD is the most common form of dementia, characterized histopathologically by extracellular amyloid-beta (A*β*) plaques and intraneural fibrillar tangles (microtubule-associated tau protein aggregates) along with synaptic and neuronal losses. These pathological processes are initiated in the entorhinal cortex and hippocampus for unknown reasons and spread as the disease progresses, resulting in cortex and hippocampal shrinkage, dementia, personality changes, and death. Late-onset (>60 years) sporadic AD accounts for most cases and has been suggested to result from complex interactions among multiple genetic, epigenetic, and environmental factors [2] where food composition, physical, and mental activities appear important [25], but for which chemical exposure risk factors other than possibly smoking [25] have been difficult to identify. Individuals carrying the mutant apolipoprotein E4 (apoE4) allele are at increased risk for late-onset AD which is believed to be related to apoE's role in A*β* clearance [2, 26]. Early-onset (<60 years) autosomal dominant genetic inheritage accounts for less than 1% of all AD cases [2] and affects individuals carrying mutations in three genes: the amyloid precursor protein (APP), and presenilin 1 and 2 (*γ*-secretase components). Unlike this genotype, a rare APP mutation positioned close to the site where *β*-secretase cleaves APP into amyloid-*β* leads to reduced formation of A*β* peptide monomers and protection against AD [27]. A*β* peptides (ca. 90% A*β*_40_ and 10% A*β*_42_, of which A*β*_42_ is considered to be more toxic) are formed through sequential intramembranous and extraneuronal proteolytic processing of the transmembranal APP by *β*- and *γ*-secretases (Figure 1). A*β* has been suggested to act as an antioxidant when present as a monomer but seems to lose this function when aggregated into oligomers or plaques, then becoming a reactive oxygen species (ROS) generator [28]. The high prevalence of sporadic AD, with an incidence of approximately one person out of twenty over the age of 65 suffering from Alzheimer's disease [29], has yet not been explained by single exposure factors. To some surprise, a moderate alcohol consumption appears to be protective against dementia [30], possibly due to anti-inflammatory effects [31].

In the light of new knowledge, we hypothesize that the iron and inflammation responsive hormone hepcidin could cause metal dyshomeostasis and oxidative stress in AD. We therefore performed *in silico* gene analyses of proteins regulating metal homeostasis and compared AD cases with unaffected elderly. Regarding environmental exposure risks, recent studies suggest that highly potent neurotoxins from food and drink contaminated with cyanobacteria can induce AD-resembling pathologies. Food constituents (e.g., antioxidants) can also protect against AD and much effort is spent on developing treatments, including drugs. These topics are discussed below.

### 2.1. Metal Dyshomeostasis in AD

For both AD and PD, numerous studies support a dysregulated metal (iron, copper, and zinc) brain homeostasis and metal catalysed oxidative damages [10–13]. A recent meta-analysis study on reported AD (versus aged controls) brain metal levels found no support for elevated neocortex iron, copper, or zinc levels, but significantly decreased neocortex copper levels when considering quantitative (metal content per wet weight tissue) analyses [32]. The same study also found a significant publication bias, with papers reporting increased iron levels were much more frequently cited than those reporting no change or decreased levels [32]. Still there is convincing support of the notion that certain A*β*-plaques forms contain iron, copper, and zinc [22–24], and individual intracellular or brain regional metal levels may also differ. Whereas A*β*-plaque associated iron and copper ions can redox cycle and produce ROS [11], zinc does not, but has been reported to be a particularly good A*β*-plaque aggregator [24, 33] and a tau hyperphosphorylation inducer [34]. Zinc may also inhibit APP's iron-export ferroxidase activity [18]. Approximately 20–40% of cognitively normal elderly people also show evidence of significant brain A*β*-plaque depositions [35] suggesting that not all plaque forms are toxic. Another recent meta-analysis found significantly increased serum copper levels among AD patients versus controls [36]. Positive outcomes dominate when metal chelators were administrated therapeutically in AD animal models and in clinical human AD studies, see below.

### 2.2. Are Hepcidin and APP Causal for Decreased Copper Levels in AD Cortex Neurons?

In the brain, the complex processes regulating metal delivery to the various cell types, metal storage and export mechanisms, are yet not fully understood. In aging, a mild systemic inflammation [37] and elevated iron levels in some organs (brain [38–40], skeletal muscle [41, 42], and liver [41]) is commonly seen, while iron levels decrease in bone marrow (related to anaemia) [43]. Human brain iron levels increase with age to reach a relative steady state at around 55 years in neurologically normal subjects [32]. Hepcidin, a master regulator of body iron metabolism, is an evolutionary conserved antimicrobial-like peptide hormone expressed in several organs including brain [20, 44] as part of the innate (nonspecific) immune system in response to pathogens [45] including lipopolysaccharide (LPS) [44]. Hepcidin restricts iron availability for microbes by its binding to the only known cellular iron exporter ferroportin in host cell membranes which causes internalization and lysosomal ferroportin degradation (Figure 1) [19]. Liver hepcidin expression can be induced via the bone morphogenetic protein (BMP6) pathway by intracellular iron or through the JAK-STAT3 pathway by the cytokine interleukin 6 (IL-6; possibly also other cytokines) [46, 47]. Brain hepcidin mRNA increases with aging in mice [20]. The expression of the iron exporter ferroportin is induced by iron (and heme) through an mRNA iron regulatory element (IRE) [48]. However, in experiments studying neuronal cell (substantia nigra) produced hepcidin [49] or injected hepcidin [50], hepcidin was found to downregulate ferroportin expression, suggesting that hepcidin (over iron) dominantly controls ferroportin. Systemic increased plasma hepcidin lowers transferrin-bound plasma iron by inhibiting iron release from several organs (also liver). Reticuloendothelial cells (macrophages) accumulate iron through constant red blood cell phagocytosis; with increased hepcidin less iron passes on towards the bone marrow for red blood cell synthesis by hematopoietic cells [46], which may cause age-related anaemia [51]. Also iron absorption from food in the gut is blocked since hepcidin prevents enterocyte-mediated iron export into the blood system. It is presently unclear if liver produced hepcidin reaches the brain, but several peptides are known to cross the blood-brain barrier (BBB). It is suggested that hepcidin in the brain mainly originates from other organs (unpublished data) [52]. The same study found that hepcidin was absent in microglia but colocalized with ferroportin in neurons and astrocytes [52]. Hepcidin's inhibition of ferroportin [19] may increase intracellular neuronal iron levels which can induce APP-mRNA translation due to an IRE stem loop in the 5′-untranslated region [21].

APP is a multifunctional metalloprotein containing a copper binding domain (not localized in its A*β* part) [53]. APP was found to possess ferroxidase (oxidizes Fe^2+^ into Fe^3+^) activity assisting in plasma membrane Fe^2+^-export by ferroportin, counteracting iron accumulation and oxidative stress [18]. APP's ferroxidase activity has been shown to take place on the extracellular plasma membrane side, where APP (in interaction with ferroportin) loads Fe^3+^ into blood transferrin [18] (Figure 1). Ceruloplasmin also has ferroxidase activity, but this protein is normally not expressed in cortical neurons [54]. APP may therefore be the sole iron-exporting ferroxidase in neurons [18].

In neuronal supporting astrocytes, cellular copper export is mediated by the copper transporting P-type ATPase ATP7A, which translocates from the trans-Golgi network to the plasma membrane in the presence of elevated copper [55]. Free intracellular copper is bound to metallothioneins or is stored in vesicular copper pools, but copper levels are generally low, much lower than the iron pool which includes cytosolic and mitochondrially stored iron in the form of ferritin. We hypothesize that constitutive expression of APP may be responding to elevated iron levels in individual neurons, with the purpose of assisting iron export by ferroportin through the plasma membrane. However, since ferroportin may have been internalized by hepcidin, and since APP has a copper binding domain [53], constitutive APP transmembranal passage and extracellular proteolysis may reduce the already relatively low intracellular copper pool, resulting in suboptimal copper levels in individual neurons. In support of this (see also Section 2.3), overexpression of the APP in transgenic mice resulted in significantly lower copper levels, but the iron levels remained unaltered [56]. Enzymes requiring copper include cytoplasmic Cu/Zn-superoxide dismutase (intracellular SOD1, extracellular SOD3), tyrosinase, cytochrome c oxidase (COX), ceruloplasmin, dopamine beta-hydroxylase, hephaestin, lysyl oxidase, peptidylglycine alpha-amidating monooxygenase (PAM), and amine oxidases, of which several are important for neurological functioning [57]. COX is vital for mitochondrial respiratory ATP production, and fewer well-functional mitochondria may induce apoptosis. Significantly decreased COX activities (but not respiratory complexes I + III, II + III activities) in AD temporal cortex and hippocampus were observed [58]. Decreased SOD1 activity and increased cerebrospinal fluid copper levels were noted in several neurodegenerative diseases [59].

In addition to the described cellular challenges, microglial cells recognize and attack A*β*-plaques, starting a vicious circle of ROS (e.g., superoxide, O_2_^•−^; hydrogen peroxide, H_2_O_2_; and hypochlorous acid, HOCl) and cytokine (e.g., IL-6, IL-1*β*, and tumour necrosis factor-alfa, TNF-*α*) release [60] (Figure 1). ROS is also produced from extracellular iron and copper ions attached to A*β*-oligomers and plaques [23, 24], and peroxides react with loosely attached redox-cycling intracellular iron ions attached to biomolecules (e.g., nucleic acids, lipids, and proteins) inflicting oxidative damage. Elevated intracellular ROS levels can also be sensed by transcription factors such as heme oxygenase-1 (HO-1) [61] and also nuclear factor-kappa B (NF-*κ*B) [62], which can lead to further production of inflammatory cytokines also in neurons. Notably, microglia also have a protective role by mediating clearance of A*β* through phagocytosis [63].

Negatively charged nucleic acids attract loosely attached iron ions inflicting ROS mediated damages through Fenton chemistry [64, 65]. In particular, oxidative RNA damages, commonly observed in AD [10, 66, 67], may cause erroneous protein translations or truncations leading to dysfunctional proteins [66]. Observation of significantly less protein in AD frontal cortex has been reported [68]. Reactive byproducts from lipid peroxidation include the intermediates 4-hydroxy-2,3-nonenal (HNE), 4-oxo-2-nonenal (ONE), and acrolein, that react with macromolecules forming alkylative adducts [12, 69]. Metal-catalysed oxidation of amino acids generates protein carbonyls [70, 71] which may hinder proper protein function. Not all forms of alkylative and oxidative lesions are well repaired. 

### 2.3. *In Silico* Analysis of Genes Involved in Brain Copper/Iron Homeostasis Pathway

**Figure 2:**
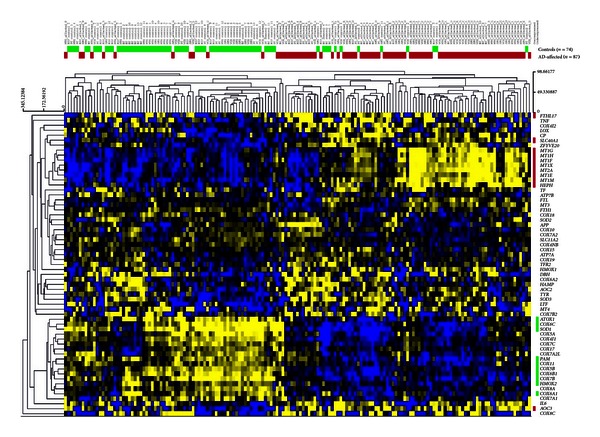
Unsupervised hierarchical clustering analysis of 61 selected genes involved in brain copper/iron homeostasis, comparing gene expression profiles of AD and unaffected age-matched controls. Unsupervised hierarchical clustering analysis (complete-linkage and Manhattan distance similarity measurement) is based on similarities in gene expression. The 21 SAM-identified genes are color coded (right side of figure) based on the group they belong to: red color code represents 11 overexpressed genes, while green color code represents 10 underexpressed genes. The horizontal red color bar indicates AD-affected samples (*n* = 87), whereas the green bar indicates normal elderly controls (*n* = 74).

We wanted to investigate how the expression patterns of genes involved in brain copper/iron homeostasis pathway are modulated in neurologically normal (healthy) elderly and AD-affected individuals. To do so, we performed a search in the publicly available gene expression database NCBI Gene Expression Omnibus (GEO) [72]. We used the microarray data from Liang et al. (2008) [73] and Liang et al. (2007) [74] (GEO accession number GSE5281), which are comprehensive genome-wide gene expression studies of samples collected from six brain regions that are either histopathologically or metabolically relevant to AD: hippocampus (HIP), entorhinal cortex (EC), middle temporal gyrus (MTG), posterior cingulate cortex (PC), superior frontal gyrus (SFG), and primary visual cortex (VCX) [73, 74]. For more detailed description of sample collection, experimental design and flow, we refer the reader to the original studies [73, 74]. We downloaded the raw microarray data (GSE5281) deposited in the GEO database [72]. The raw microarray data were reanalyzed using J-Express v2009 as described previously [75, 76]. From the dataset we selected 61 genes involved in brain copper/iron homeostasis pathway. The processed intensities of the selected 61 genes were log2-transformed.  Figure 2 shows unsupervised hierarchical clustering analysis of these 61 genes, and the results were visualized in a dendrogram using the MeV v4.7 software [77]. By visual inspection of the heatmap (Figure 2), we observed that samples from AD-affected individuals clustered close to each other in one branch while samples from normal elderly individuals clustered in the other branch. Two-class, unpaired SAM (Significance Analysis of Microarray) [78] analysis was conducted in order to identify genes whose mean expression level is significantly different between AD-affected and normal elderly individuals control samples. Twenty-one genes were differentially expressed (false discovery rate, FDR < 10%). Eleven of these genes were overexpressed in AD-affected individuals and underexpressed in normal elderly individuals, whereas 10 genes were underexpressed in AD-affected individuals and overexpressed in normal elderly individuals (Table 1). Genes overexpressed in AD-affected individuals include *FTHL17, SLC40A1, AOC3, MT1E, HEPH, MT1X, MT1H, MT1F, MT1G, MT2A,* and *MT1 M *(Table 1), and most of these genes clustered close to each other in one branch (Figure 2), while *COX6A1, COX6C, HMOX2, COX11, SOD1, PAM, ATOX1, COX5B, COX6B1,* and *COX7B* were underexpressed (Table 1), and clustered together in another branch (Figure 2). Notably, brain tissue is composed of several types of cells (astrocytes, neurons, microglia, and endothelial cells, etc.) where neuron supporting astrocytes numerically dominate, but the results show that crucial copper-dependent enzymes (COX, SOD1, and PAM) and ATOX1 (a copper-chaperone protein) are underexpressed in AD brains. Amine oxidase (copper containing 3), however, was overexpressed in AD brains for unclear reasons. Moreover, in AD brains, several metallothioneins (small copper and zinc binding proteins) are overexpressed, as well as iron binding ferritin and ferroportin (SLC40A1). Hephaestin (contains copper but functions as an iron ferroxidase in collaboration with ferroportin) was overexpressed in AD brains. Tight transcriptional control of the Cu/Fe homeostasis pathway involved genes in the brain is very important, and dysregulation of these genes might affect the Cu/Fe balance, resulting in potential damage to brain function. The gene expression profiles constituted by these altered genes can be used to identify candidate genes to be associated with AD, which might contribute to early detection of this complex disorder. Identifying AD-predictive genes may uncover gene products with mechanistic properties relevant to AD; this should be pursued in future studies.

### 2.4. Cyanobacterial Toxins-Potential Tauopathy and AD Risk Factors

Cyanobacteria and their potential impact on human health are an emerging public health issue that has received increasing scientific interest resulting in new research [79–81]. While it has been confirmed that cyanobacteria produce toxins (e.g., microcystins, MCs [80], and chemicals resembling amino acids [81]) that are potentially capable of causing neurological disorders in humans, many questions remain unanswered regarding the identification and quantification of cyanobacteria and cyanotoxins in the environment and to what degree they translate into health risks.

**Figure 3:**
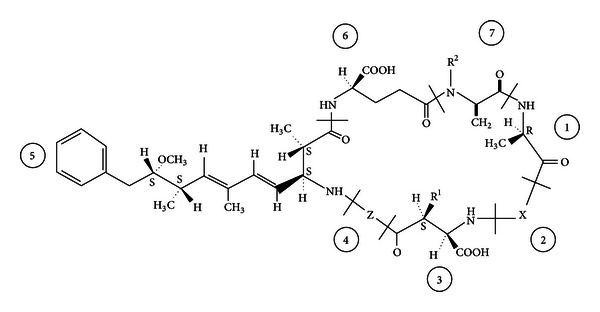
General “cyclo–(D-Ala^1^–X^2^–D–MeAsp^3^–Z^4^–Adda^5^–D–Glu^6^–Mdha^7^)” structure of microcystins (MCs), showing the most frequently found variations. X and Z are variable L-amino acids (in MC-LR, MC-LF and MC-LW, X = L-Leucine (L) and Z = arginine (R), phenylalanine (F) or tryptophan (W)); R^1^ and R^2^ are H (demethylmicrocystins) or CH_3_; D-MeAsp is D-*erythro*-*β*-methylaspartic acid and Adda is (2*S*,3*S*,8*S*,9*S*)-3-amino-9-methoxy-2,6,8-trimethyl-10-phenyldeca-4,6 dienooic acid; Mdha is N-methyldehydroalanine (Dha = dehydroalanine). From [82].

Several freshwater cyanobacteria produce MCs that irreversibly inhibit serine/threonine-specific protein phosphatases [83] and have caused morbidities in animals and humans [84, 85]. In a severe human MC intoxication 1996 in Caruaru, Brazil, patients developed signs of acute neurotoxicity, for example, deafness, tinnitus, intermittent blindness, as well as subsequent hepatotoxicity [86]. In conjunction with some animal studies this suggests that MCs are potent neurotoxins acting by inducing caspase activity, chromatin condensation, and microtubule tau hyperphosphorylation [79, 83]. In one study, the effect of MCs on neurite degeneration has been analyzed with confocal microscopy; neurite length was determined using image analysis [83]. MC induced significant neurodegeneration already at 0.5 *μ*M MC-LF (Figure 3) and neuronal apoptosis was significantly increased by the MC variants MC-LF and MC-LW at higher concentrations (≥3 *μ*M). Moreover, sustained hyperphosphorylation of the tau protein with all MC congeners was found [83]. The concentration and congener-dependent mechanisms observed suggest that low concentrations of MC-LF and MC-LW can induce subtle neurodegenerative effects, reminiscent of Alzheimer's disease type human tauopathies. Such effects should be taken more seriously with regard to potential human health effects, than the apical cytotoxicity (apoptosis or necrosis) demonstrated at high MC concentrations [83]. It has been shown that MC-LR (see Figure 3) treated hippocampi showed alterations in proteins involved in cytoskeleton, neurodegenerative disease, oxidative stress, apoptosis, and energy metabolism; three proteins related to neurodegenerative disease, septin5,  *α*-internexin, and  *α*-synuclein, were identified to be altered by MC-LR exposure. It was found that MC-LR induced inhibition of protein phosphatases and abnormal hyperphosphorylation of the neural microtubule associated protein tau [79]. This was found to lead to impairment of learning and memory, accompanied by severe damage and neuronal apoptosis in the hippocampal CA1 regions of rats. Thus, MC-LR was found to induce tau hyperphosphorylation, spatial memory impairment, neural degenerative changes, and apoptosis, suggesting that this cyanotoxin may contribute to Alzheimer's disease in humans [79].

**Figure 4:**
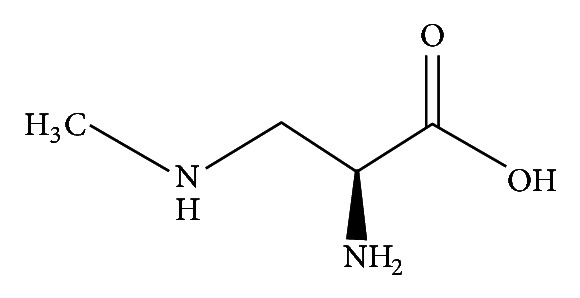
*β*-Methylamino-L-alanine (BMAA).

Cyanobacteria also produce beta-methylamino-L-alanine (BMAA; Figure 4), a nonprotein amino acid produced by several cyanobacteria including *Spirulina* that can be misincorporated into protein chains within human neurons, causing proteins to misfold and form aggregates within the cells [87] and is thought to lead to neurofibrillary tangles, an indication of neurodegenerative disease. Whereas a tie between cyanobacteria and human health is rather well accepted [80], at this point there is interestingly also a possible tie between cyanobacterial toxins and risk of progressive neurodegenerative disease (BMAA was identified in neuroproteins from AD and amyotrophic lateral sclerosis (ALS) brains [88]), an issue which is rarely discussed.

Exposure to cyanobacteria toxins can occur by drinking untreated water from a lake, pond, reservoir, or spring with a cyanobacterial bloom, or when ingesting contaminated foods (e.g., crops watered with untreated water) [89]. Also aerosol inhalation from blooming waters may pose a health risk, for example, when watering the lawn. MC intoxication frequently occurs in conjunction with toxic cyanobacterial blooms in water reservoirs (lakes and ponds) used for drinking water or recreational purposes. It has also been suggested that nutritional supplements from cyanobacteria (blue-green algae) such as *Spirulina* and *Aphanizomenon* may not only be beneficial as they may contain MCs which constitute a group of cyanobacterial toxins with more than 100 congeners [90]. Since cyanobacteria blooms occur all over the world, the possible implication of the cyanobacteria for health problems might be severe. Globally the most frequently found cyanobacterial toxins in blooms from fresh and brackish waters are the cyclic peptide toxins of the microcystin and nodularin family [82]. Large numbers of the human population are therefore at risk to be exposed to these toxins with adverse health effects.

### 2.5. Treatment of and Protection from AD

Removing pathogenic A*β* peptides to prevent, and possibly reverse, aggregation is the most sought after method for slowing or delaying the onset of AD. Development of AD-modifying drugs include amyloidtargeting monoclonal antibodies (mAbs), *β*- and *γ*-secretase inhibitors, anti-amyloid vaccines, and tau-based therapies [3]. In principle, A*β* targeting mAbs should penetrate the blood-brain barrier and recognize various forms of A*β*, guiding A*β* clearance into the blood stream for liver degradation. However, some recent clinical mAbs phase III trials have failed [3]. Secretase inhibitors are intended to decrease APP cleavage and lower the amount of A*β* peptides formed, and, vaccines can be used to boost the immune system to recognize A*β*. Tau-based therapies are reported to moderate downstream effects due to A*β*-plaque build-up [3].

The hepcidin-mediated copper lowering mechanisms discussed above suggest that locally active antagonists interfering with hepcidin's binding to neuronal cell's ferroportin would be expected to promote neuronal ferroportin export of iron, decreasing undesired APP-mediated copper export as well as iron-mediated oxidative damages intracellularly. Ways of downregulating hepatic hepcidin expression (e.g., siRNA) could be attempted, but lowered systemic hepcidin levels will also result in increased intestinal iron absorption. Membrane-permeable metal chelators capable of washing out toxic metals may be effective [91–94], and *in vitro*, A*β* deposits can be dissolved using metal chelators [22]. Lowering systemic iron levels by controlled phlebotomy has been suggested as an AD therapeutic [95]. A clinical study administrating deferoxamine (a nonmembrane-permeable strong Fe^3+^-chelator that to some degree is taken up into cells by endocytosis, but which does not cross the BBB readily) slowed the clinical progression of dementia associated with AD [96]. For a patient suffering from an iron metabolism disorder, treatment for 10 months with the iron chelator deferoxamine decreased brain iron stores, prevented progression of the neurological symptoms, and reduced plasma lipid peroxidation [97]. Many issues confront the use of chelators including metal saturation. The compounds' ability to reach the intended site is affected by metabolic instability, and toxicity is an important issue (administration of copper chelators to dissolve A*β*-plaques could further lower neuronal copper levels, etc). Current clinical oral administration of lipophilic membrane permeable iron chelators includes deferiprone (produced by ApoPharma) and deferasirox (Novartis). These are today mainly used for treatment of thalassemia major and anaemia, respectively; only a few reports concern their uses in relevant neurodegenerative situations [98]. Molecular modifications may be needed for improved BBB passage and stability [99]. For efficient iron removal from the brain, it has been suggested that the lipophilic chelator-iron (III) complex should have a molecular mass less than 500 Da and be uncharged for cell membrane and BBB permeability [93]. The copper/zinc chelator clioquinol (CQ) [100] was suggested to inhibit A*β* accumulation due to its ability to remove metals from the brain; however, CQ is now considered toxic. This led to the development of the analogue PBT2, a potential therapeutic compound for AD which reduced cerebrospinal fluid (CSF) A*β*_42_ and improved cognition without serious adverse effects, according to a phase II clinical trial [101]; the compound is being further investigated. Other chelators tested in AD models having showed positive effects include the iron chelator M30 [92], the copper chelators JKL-169 [102] and IQM-622 [103], and the calcium/copper/zinc chelators DP-109 and DP-460 [104, 105]. On the contrary, a clinical trial of D-penicillamine, a copper chelator, was unable to produce any clinical improvement in AD patients and resulted in toxicities [106]. Nanoparticle-based chelators that can cross the blood-brain barrier are also being developed [107].

Antioxidants from food such as flavonoids, carotenoids, tocopherols, and selenium have been suggested to protect against AD [94, 108–111]. Interestingly, the occurrence of AD in India was found to be just one quarter of that in the USA which may be related to intake of the plant phenolic antioxidant curcumin possessing iron binding capacity [112]. Curcumin is present in roots of the turmeric plant used as spice in various foods including curry [108, 113]. Natural antioxidants have also showed protective effects against PD [114], and can also lower microglial cytokine production [115]. Also food compounds not expected to act as antioxidants may protect against neurodegeneration. One example is caffeine (present in coffee) which has been suggested to be a potent neuroprotector [116, 117]. 

## 3. Parkinson's Disease (PD)

PD is today recognized as the second most common neurodegenerative disorder after AD. The disease is known to cause motor dysfunctions in addition to autonomic and cognitive deficits [118]. PD is characterized by the accumulation of  *α*-synuclein and selective and progressive loss of dopaminergic neurons in the substantia nigra (SN) and the degeneration of projecting nerve fibres in the striatum [119, 120]; however, its true aetiology still remains unknown.  *α*-Synuclein aggregation is thought to appear early in disease development and to spread across the nervous system as the disease progresses [121], and early neuroimmune activation by oligomerized  *α*-synuclein has been speculated to precede neuronal degeneration. There are probably both environmental and genetic (familial) causative factors for developing PD. About 5–10% of patients are known to have monogenic forms of the disease; for those, mutations in at least 13 loci and 9 genes have been reported to correlate with PD [122]. However, exposure to environmental factors may play a more significant role than genetic mutations in the vast majority of PD patients (sporadic PD) [123, 124]. This is supported by an etiology study of twins with PD [124], where it was found that genetic factors do not play a major role in causing typical PD; no genetic component was evident when the disease began after the age of 50. On the other hand, genetic factors appear to be important when the disease began at or before the age 50 [124]. It is then tempting to speculate that aging may play a predominant role in the etiology of PD but in combination with environmental toxins. 

Hypotheses on the loss of dopaminergic neurons in PD suggest that mitochondrial dysfunction may be involved in neuronal death [125, 126], through mechanisms involving oxidative stress and impaired energy metabolism [127, 128]. Inhibition of complex I can lead to ROS, lipid peroxidation, protein dysfunction, and a decrease in reduced glutathione: all hallmark features of postmortem PD [129]. Moreover, chemically induced mitochondrial dysfunction and cellular lesions can activate inflammatory pathways. Below we discuss the role of inflammation in the context of environmental risk factors for the development of PD. We also present data from *in silico* analysis of published transcription data of PD compared to normal brains.

### 3.1. Inflammation in PD

Inflammation in the brain has recently garnered much interest due to clinical investigations, which show an increase in activated microglia and high levels of inflammatory factors in the nigrostriatal system from postmortem analyses of PD patients [130]. In some cases, the onset of PD has been reported to be associated with head trauma or encephalitis, also suggesting that an inflammatory component is involved in the disease process [131]. Levels of proinflammatory cytokines are elevated in PD patients compared with healthy subjects, and it has been shown that these cytokines can contribute to dopaminergic cell death *in vivo* [132, 133]. Indeed, several recent studies have demonstrated an important inflammatory component in PD pathogenesis. Injection of lipopolysaccharide (LPS), an endotoxin produced by gram-negative bacteria, into the SN [134] or striatum [135] induces inflammation in the brain, and this has been used as a tool to produce animal models of PD. As reported by Zhou and coworkers in 2012, a single intracerebroventricular injection of LPS led to activation of microglia in the hippocampus, striatum, and in the SN, followed by phospho-*α*-synuclein expression and abnormal motor behaviour [134]. Interestingly, these results indicate that microglia are activated for several months after a single, low dose injection of LPS in the rat, which eventually results in progressive and selective damage to dopaminergic neurons in the SN.

Although PD and AD have distinct pathologies, they also share some similarities. Both underlying pathologies present different forms of protein aggregates that appear to be causally linked to their respective diseases [151]. The pathological hallmark of AD involves misfolding and aggregation of A*β*-plaques and neurofibrillar tangles, mainly composed of the protein tau, whereas PD inclusions (Lewy bodies) are largely composed of intracytoplasmic aggregates of  *α*-synuclein. However, aggregations of A*β* and  *α*-synuclein are not unique to AD and PD; the pathologies are often found to coexist in patients with each of these conditions [152, 153]. *  α*-Synuclein has been reported to activate microglia in the SN pars compacta of mice, which precipitates dopaminergic degeneration [154]. *In vitro* observations have suggested that  *α*-synuclein can have a direct effect on microglial activation [155]. An increased susceptibility of dopaminergic cells to LPS-induced toxicity is seen in neurons overexpressing the human form of  *α*-synuclein, and the generated inflammatory response further leads to aggregation of  *α*-synuclein in nigral neurons [156]. Furthermore, A*β* deposition may be deleterious not only in AD but also in PD, and this A*β* effect is associated with inflammatory processes. Inflammation can promote A*β* deposition that can in turn lead to ROS formation, neuronal cell death [157], glia cell activation, and subsequent release of proinflammatory cytokines [158, 159]. Hochstrasser et al. (2013) showed that A*β* significantly decreased the number of, for example, hydroxylase-positive dopaminergic neurons, and that anti-inflammatory drugs partially counteracted the A*β*-induced neuronal decline through the suppression of glial cell activation [160]. Recent data have indicated that A*β* is slightly decreased in the cerebral spinal fluid of PD patients and A*β* can interact with *α*-synuclein to accelerate cognitive decline [161]. Further investigations on the relation between  *α*-synuclein and A*β* may provide new insights into the molecular mechanisms of the pathogenesis of PD and suggest potential new approaches to its treatment.

### 3.2. Environmental Toxins, Microglia, and Neurodegenerative Diseases

In the brain, microglia perform dynamic cellular functions that include synaptic plasticity [162], cleaning of cellular debris, wound healing through alternative activation [163, 164], and innate immune defence. Microglial cells comprise approximately 10–12% of the cells in the brain and dominate in the grey matter, with particularly high concentrations in the hippocampus, hypothalamus, basal ganglia, and SN [165–167]. In general, age-related priming of microglia in the brain plays a role in the development of age-related inflammatory diseases [168], as shown by a progressive increase in the expression of microglial MHCII in rodents and primates [169]. A variety of noxious compounds released from inappropriately activated microglia, including ROS, reactive nitrogen species (RNS; e.g., nitric oxide, NO^•^, and peroxynitrite, ONOO^−^), proinflammatory cytokines (e.g., TNF*α*, IL-1*β*, and IL-6), and prostaglandins may be important mediators of dopaminergic cell death [170–172], which may propagate into a disease state [173]. Stressed dopaminergic neurons are reported to cause microglial activation by releasing stimulatory signalling molecules such as  *α*-synuclein, neuromelanin, and matrix metalloproteinase-3 [174–176]. Also, cytokines, such as TNF*α*, IL-1*β* [177], IL-4 [178] and IL-13 [179], are reported to initiate microglial NADPH oxidase activation and O_2_^•−^ generation.

**Figure 5:**
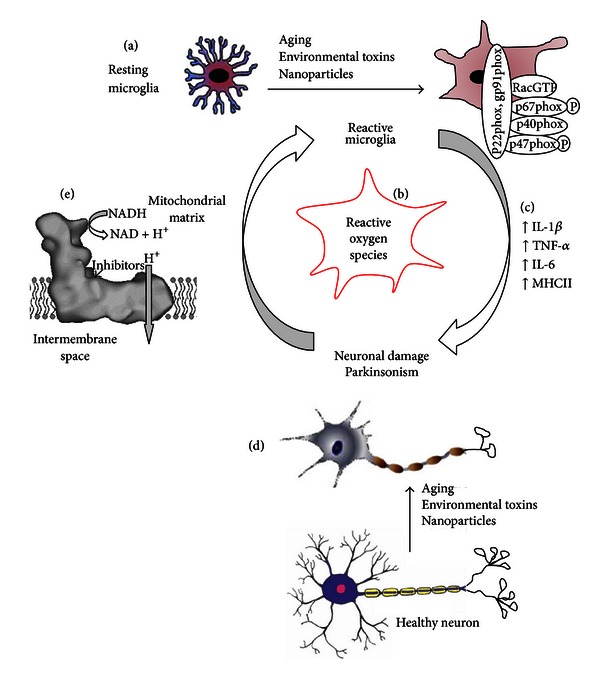
Chemical stress and aging can alter microglia reactivity and activation of the phagocytic NADPH oxidase complex. (a) Stimulation of microglia induces the parallel activation of oxidase components within the cytoplasm. This activation causes the conversion of Rac into an active GTP-bound form and the phosphorylation of p47phox and p67phox. These subunits then translocate to the membrane where they interact with p22phox and gp91phox (NOX) to initiate ROS production (b) [180]. Excessive or prolonged inflammation (e.g., IL-1*β*, IL-6, TNF-*α*) (c) and ROS resulting from increased microglial activation may contribute to neuronal damage (d). In addition, chemicals can damage complex I in mitochondria ((e) figure adapted from [181]) and induce deleterious changes to neurons in SN by ROS formation and ATP depletion. The figure is adapted with modifications from [168].

Microglia express the phagocyte NADPH oxidase [182, 183], composed of four regulatory cytosolic components (p47phox, p67phox, p40phox, and rac proteins) and a transmembrane flavocytochrome heterodimer consisting of the p22phox and the gp91phox (NOX2) catalytic subunit (Figure 5). NOX2 redox signalling has been shown to play an essential role in the microglial proinflammatory response and associated neurotoxicity, and there is evidence for ROS involvement in regulation of NOX2 subunits Rac1 and P47phox [136, 184]. Environmental toxins (including paraquat, rotenone, dieldrin, diesel exhaust particles, lindane, mancozeb, maneb, in addition to some metals; Table 2) have been reported to reach the brain and activate microglial NADPH oxidase to produce ROS and induce mitochondrial dysfunction. Continued neuronal damage and rotenone have been shown to synergistically activate NOX2 and amplify the microglial proinflammatory response to LPS and neurotoxicity *in vitro* [8, 138]. It has recently been shown that 1-methyl-4-phenyl-1,2,5,6-tetrahydro-pyridine (MPTP) intoxication is accompanied by a strong microglia response which is characterized by the release of inflammatory molecules that are believed to further drive inflammation-mediated degeneration of DA neurons [185]. Interestingly, paraquat is shown to be a potent inducer of microglial activation; both *in vivo* [186] and *in vitro* [187] studies suggest that this is the primary mechanism of dopaminergic neurotoxicity. Rotenone is known to inhibit the transfer of electrons from iron-sulphur centres in complex I to ubiquinone (coenzyme Q10). *In vitro* experiments indicate that rotenone is selectively toxic to dopaminergic neurons only in the presence of microglia [138]. Thus, altogether these studies show that triggering of microglial NADPH oxidase activation include both environmental toxins and central nervous system (CNS) disease pathways, suggesting that the microglial NADPH oxidase may be a promising target for PD treatment, especially in delaying the progression of PD.

Epidemiological studies have suggested exposure to various pesticides as risk factors for PD. These include the fungicide maneb which contains manganese [188], paraquat, and other herbicides or other pesticides [189, 190]. Chronic occupational exposure to manganese or copper, individually, or in combination with lead, iron and copper, is associated with PD [147]. In PD patients, redox-active iron and copper are considered to be an important factor in the pathology and progression of PD due to its ability to generate free radicals and to promote redox reactions. Accumulation of iron is frequently observed in brain areas linked to PD [191]. The brain regions responsible for motor functions seem to have more iron than nonmotor related regions also explaining why movement disorders such as PD are often associated with iron loading [192]. High content of copper and zinc is also present in the cerebrospinal fluid of patients affected by PD [193]; the concentrations of these metals were up to threefold higher than control levels. A detailed review of Cu, Zn, and Fe related to neurodegeneration in PD can be found in Kozlowski et al. (2012) [194]. 

Recent studies have revealed a possible link between nanoparticle (NP) exposure, and neuronal inflammation and disease. Silver (Ag) particles are one of the most used NP in consumer products; exposure has been suggested to induce learning and memory deficits in rats, possibly through oxidative stress-induced pathological changes in the hippocampus and attenuation of long-term potentiation [198]. It has previously been shown that a single intravenous exposure of rats to AgNP caused time- and size-dependent accumulation of NPs in the brain [199]. Further, AgNP induced DNA-strand breaks, apoptosis, necrosis, and decreased proliferation in different cell models [200, 201]. AgNPs may disturb the neurotransmitter signaling in the brain [202], and according to the authors such disturbances are linked to conditions such as PD and AD, and have been implicated in motor, cognitive, and affective functions. In general, the observed effects of NP in the brain can be categorized as inflammation and oxidative stress, which may be responsible for cognitive deficits and other diseases [203]. Recently, several types of inorganic NPs have been shown to activate microglia and damage neurons *in vitro* [204]. While the accumulation of NPs in brain has been clearly demonstrated, less information is available concerning potential contribution of health-related outcome(s) from NP exposure in airborne emissions, particularly in modest polluted areas. Elder et al. (2006) showed increases in proinflammatory mediators and markers of oxidative stress and immune cell activation (e.g., TNF-*α*), and SOD in rat brain regions where manganese accumulated following manganese oxide NP exposure [205]. These findings suggest that NP exposure resulted in CNS oxidative stress and inflammation. Although mechanisms driving NP-induced CNS pathology are poorly understood, new evidence suggests that microglial activation may be a key component, with an important contribution of conditions that predispose the individual to oxidative stress [206]. However, further research should address the mechanisms of toxicity and the risk of the general population for developing PD after NP exposure. Motor, cognitive, and behavioral functions have been assessed in healthy children and elderly residents in Valcamonica, Italy, and an increased prevalence of Parkinsonism was observed as being associated with the manganese levels in the deposited dust from ferroalloy airborne emissions [148]. Inflammation of the olfactory bulb and deficits in olfaction has been observed in Mexican children residing in highly polluted areas [207]. Olfactory loss is an early finding in both AD and PD and precedes cognitive and motor symptoms [208, 209].  *α*-Synuclein neuronal aggregation and accumulation of 3-nitrotyrosine and 8-hydroxydeoxyguanosine, biomarkers of oxidative stress, were also detected in nuclei of these children's brainstem [150]. That NPs can significantly enhance the rate of protein fibrillation adds a new important aspect to NP's role in neurodegeneration [207].

### 3.3. *In Silico* Analysis of Genes Involved in Parkinson's Disease

**Figure 6:**
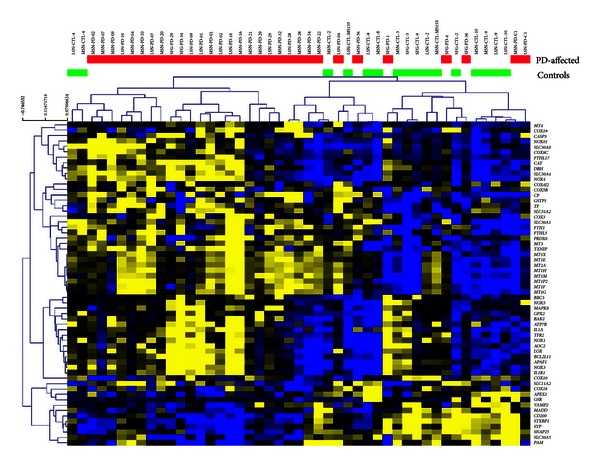
Unsupervised hierarchical clustering analysis (complete-linkage and Manhattan distance similarity measurement) of the expression of 60 selected genes in PD. The clustering analysis is based on similarities in gene expression. The yellow color code represents overexpressed genes (*n* = 47), while blue color code represents underexpressed genes (*n* = 13). The horizontal red color bar indicates PD-affected samples (*n* = 31) and the green bar indicates normal elderly controls (*n* = 16).

To further elucidate the mechanisms for degeneration of the SN in PD patients, we performed a search in the publicly available databases and in the literature for genes involved in brain metal homeostasis pathways, neurodegenerative disorders related genes (in particular PD), oxidative phosphorylation, oxidative stress, and apoptosis pathways related genes. We selected 138 genes from these pathways. To investigate whether the expression patterns of those genes are modulated in neurologically normal elderly and PD individuals, we downloaded the microarray data (GSE8397) deposited in the GEO database [72] where tissue samples from PD-cases were compared to controls [195]. Whole genome expression profiling of studies of 47 tissue samples collected from brain regions relevant to PD were analyzed for PD-cases and controls: lateral substantia nigra (LSN), medial substantia nigra (MSN), and superior frontal gyrus (SFG) [195]. For more detailed description of sample collection, experimental design and flow, we refer the reader to the original studies [195]. From the GSE8397 dataset, we identified 60 genes (out of our 138 gene list) whose mean expression level is differentially expressed between PD-cases and controls using two-class, unpaired SAM (FDR < 10%) [78]. The processed intensities of the 60 genes were mean-centered and log2-transformed.  Figure 6 shows unsupervised hierarchical clustering analysis of these 60 genes, and the results were visualized in a dendrogram using MeV v4.8 software [77]. By visual inspection of the heatmap (Figure 6), we observed that samples from PD-cases clustered close to each other in one branch while samples from controls clustered in the other branch. We further compared the identified 60 genes with gene expression datasets available in the Parkinson's disease database (Park DB: http://www2.cancer.ucl.ac.uk/Parkinson_Db2/index.php) in order to identify key genes with consistent expression profiles across several datasets. Of the 60 genes, we selected 18 genes showing significant differences across three different datasets (Table 3) [195–197]. 

Genes consistently overexpressed in PD-cases include *MT1E, MT1F, MT1G, MT1H, MT1 M, MT1P2, MT1X, MT2A, MT4, BCL2L11, CAT, SLC30A1, *and* TF*, while *CD200, SLC30A9, SNAP25, STXBP1, *and* SYP *were underexpressed (Table 3). Noticeably, in the PD brains, several metallothioneins are overexpressed, as well as iron binding transferrin. Dysregulation of these genes might disturb or compensate a dysregulated metal homeostasis. Downregulation of proteins in SN or prefrontal cortex associated with synaptic functions in neurons, such as synaptosomal-associated protein (SNAP25), synaptophysin (SYP), and syntaxin binding protein 1 (STXBP1), indicates synaptic functional disturbance and neuronal loss. Decreased CD200 expression indicates loss of neuronal regulation of microglia, and increase of CAT expression level may indicate oxidative stress. Interestingly, several genes (*NOX1, NOX3, NOX4, *and* NOX5*) coding proteins in the NADPH oxidase complex are overexpressed (Figure 6) in the PD-cases which may indicate formation of ROS and proinflammatory mediators in the SN. Altogether the expression profile constituted by these genes may serve as a basis for identification of PD-predictive genes that may explain the underlying molecular mechanisms associated with PD. This should be pursued in future studies.

## 4. Concluding Remarks

More studies are needed to investigate hepcidin's role in the brain, focusing on aging, metal dyshomeostasis, and inflammation in degenerative diseases. However, to unravel the disease aetiologies of AD and PD in relation to metal storage and shuttling between the various types of brain cells (e.g., astrocytes, neurons, endothelial cells, and microglia) new studies are required. These should, however, have high resolution and the capability to analyse protein (and/or gene) expressional and functional changes of individual cell types during disease progression. The role of combined chemical exposures for development of neuronal diseases needs to be elucidated in future studies.

## Figures and Tables

**Table 1:** Genes (*n* = 21) involved in brain metal homeostasis pathways for which expression is significantly changed in AD.

Gene symbol	Description	SAM score (*d*)*	Fold up- or downregulation
*MT1M *	Metallothionein 1M	6.7	2.50
*MT2A *	Metallothionein 2A	5.3	2.00
*MT1G *	Metallothionein 1G	4.8	2.30
*MT1F *	Metallothionein 1F	4.6	2.30
*MT1H *	Metallothionein 1H	4.4	2.00
*MT1X *	Metallothionein 1X	4.2	1.80
*HEPH *	Hephaestin	4.1	2.00
*MT1E *	Metallothionein 1E	4.0	1.80
*AOC3 *	Amine oxidase, copper containing 3 (vascular adhesion protein 1)	3.8	2.90
*SLC40A1 *	Solute carrier family 40 (iron-regulated transporter), member 1	3.0	1.50
*FTHL17 *	Ferritin, heavy polypeptide-like 17	2.2	1.60
*COX6A1 *	Cytochrome c oxidase subunit VIa polypeptide 1	−3.3	−1.43
*COX6C *	Cytochrome c oxidase subunit VIc	−3.7	−1.67
*HMOX2 *	Heme oxygenase (decycling) 2	−3.7	−1.67
*COX11 *	COX11 cytochrome c oxidase assembly homolog (yeast)	−4.3	−1.43
*SOD1 *	Superoxide dismutase 1, soluble	−4.5	−2.00
*PAM *	Peptidylglycine alpha-amidating monooxygenase	−4.6	−1.67
*ATOX1 *	ATX1 antioxidant protein 1 homolog (yeast)	−4.6	−2.00
*COX5B *	Cytochrome c oxidase subunit Vb	−5.2	−1.67
*COX6B1 *	Cytochrome c oxidase subunit VIb polypeptide 1 (ubiquitous)	−5.3	−2.00
*COX7B *	Cytochrome c oxidase subunit VIIb	−5.8	−2.00

*Significantly differentially expressed genes between AD-affected cases and controls, FDR < 10% for GSE5281 microarray date, and unlogged fold up- and downregulated genes (*n* = 21). Eleven genes were overexpressed and 10 genes were underexpressed in the samples from AD-affected cases in comparison to age-matched unaffected controls.

**Table 2:** Environmental toxins linked to neuronal diseases.

Environmental toxins (linked to brain disease)	Implicated disease/condition in humans or in animal studies	References
Cyanobacterial toxins (MCs, BMAA)	Tau hyperphosphorylation in AD (MC) and protein misfolding (BMAA)	[79, 83, 87]
Smoking	Dementia, AD	[25]
Paraquat	Cell death dopaminergic neurons (Bcl-2 induced) and PD possibly by mitochondrial dysfunction and oxidative stress	[6, 136, 137]
Rotenone	Oxidative stress, potent inhibitor of mitochondrial complex I, nigrostriatal cell death (Bcl-2 induced) and PD	[8, 138]
Dieldrin	PD	[139]
Diesel exhaust particles (air pollution)	PD	[140]
Lindane	PD	[141]
Mancozeb	PD	[142]
Maneb	Oxidative stress, cell death (Bcl-2 induced), and PD	[6, 142]
3-nitropropionate	Inhibits succinate dehydrogenase; striatal degeneration; Huntington's disease	[143, 144]
Trichloroethylene	Mitochondrial dysfunction in striatum and PD	[145]
1-methyl-4-phenyl-1,2,5,6-tetrahydropyridine (MPTP)	Oxidative stress, cell death (Bcl-2 induced), and PD	[7]
Insecticides (Lorsban, Dursban, or other chlorpyrifos products)	PD	[146]
Metals (e.g., manganese, copper)	PD	[147–149]
Nanoparticles in air emissions	Brain functional deficits?	[150]

**Table 3:** Genes (*n* = 18) involved in brain metal homeostasis and neurodegeneration for which expression is significantly changed in PD.

Gene symbol	Description	SAM score (*d*)*	Fold up- or downregulation	Fold change^a^	Fold change^b,c^
*MT1G *	Metallothionein 1G	1.7	1.94	0.90	0.82
*MT1F *	Metallothionein 1F	1.6	1.87	0.88	0.55
*MT1M *	Metallothionein 1M	1.5	1.73	1.10	0.92
*MT1P2 *	Metallothionein 1 pseudogene 2	1.5	1.79	0.43	0.57
*MT1H *	Metallothionein 1H	1.5	1.65	0.69	0.79
*MT1E *	Metallothionein 1E	1.5	1.64	0.62	0.58
*MT1X *	Metallothionein 1X	1.4	1.56	0.67	0.73
*BCL2L11 *	BCL2-like 11 (apoptosis facilitator)	1.4	2.05	0.29	0.61
*MT2A *	Metallothionein 2A	1.3	1.51	0.46	0.81
*SLC30A1 *	Solute carrier family 30 (zinc transporter), member 1	1.3	1.38	0.47	0.37
*MT4 *	Metallothionein 4	1.2	3.51	0.29	0.22
*CAT *	Catalase	1.1	2.00	0.30	0.40
*TF *	Transferrin	1.0	1.62	0.68	0.74
*SNAP25 *	Synaptosomal-associated protein, 25 kDa	−1.3	−1.50	−1.50	−1.90
*SYP *	Synaptophysin	−1.3	−2.04	−0.51	−0.53
*CD200 *	CD200 molecule	−1.5	−1.90	−0.62	−1.10
*STXBP1 *	Syntaxin binding protein 1	−1.8	−1.90	−1.00	−0.71
*SLC30A9 *	Solute carrier family 30 (zinc transporter), member 9	−1.9	−1.79	−0.69	−0.63

*Significantly differentially expressed genes between PD-cases and controls, FDR < 10% for GSE8397 microarray date and unlogged fold up- and downregulated genes (*n* = 18). Thirteen genes were overexpressed and 5 genes were underexpressed in the samples from PD-affected cases in comparison to age-matched unaffected controls. The microarray dataset is from (a) microarray dataset from Moran et al., 2006 [195] with GEO accession number, GSE8397; (b) microarray dataset from Zhang et al. 2005 [196] with accession number GSE20168; (c) microarray dataset from Lesnick et al. 2007 [197] with GEO accession number GSE7621. All three microarray datasets are reanalyzed in the Parkinson's disease database (Park DB: http://www2.cancer.ucl.ac.uk/Parkinson_Db2/index.php).

^a,b,c^Are log-transformed fold change differences between PD-cases and controls from Park DB.
